# Axial superior facet slope may determine anterior or posterior atlantoaxial displacement secondary to os odontoideum and compensatory mechanisms of the atlantooccipital joint and subaxial cervical spine

**DOI:** 10.1007/s00330-023-09544-w

**Published:** 2023-03-22

**Authors:** Yan Chen, Han Du, Xiaofei Cheng, Jie Zhao, Han Qiao, Changqing Zhao

**Affiliations:** 1 Department of Orthopaedic Surgery, Shanghai Key Laboratory of Orthopaedic Implants, Shanghai Ninth People’s Hospital, Shanghai Jiao Tong University School of Medicine, 639 Zhizaoju Road, Shanghai, 200011 People’s Republic of China; 2 Shanghai Key Laboratory of Orthopaedic Implants, Shanghai Ninth People’s Hospital, Shanghai Jiao Tong University School of Medicine, Shanghai, People’s Republic of China

**Keywords:** Os odontoideum, Atlantoaxial displacement, Axial superior facet, Axial superior facet slope, Cervical sagittal parameters

## Abstract

**Objective:**

To introduce novel parameters in determining directions of os odontoideum (OO) with atlantoaxial displacement (AAD) and compensations of cervical sagittal alignment after displacement.

**Methods:**

Analysis was performed on 96 cases receiving surgeries for upper cervical myelopathy caused by OO with AAD from 2011 to 2021. Twenty-four patients were included in the OO group and divided into the OO-anterior displacement (AD) group and the OO-posterior displacement (PD) group by displacement. Seventy-two patients were included as the control (Ctrl) group and divided into Ctrl-positive (Ctrl-P) group and Ctrl-negative (Ctrl-N) group by axial superior facet slope (ASFS) in a neutral position. ASFS, the sum of C2 slope (C2S) and axial superior facet endplate angle (ASFEA), was measured and calculated by combining cervical supine CT with standing X-ray. Cervical sagittal parameters were measured to analyse the atlantoaxial facet and compensations after AAD.

**Results:**

Atlas inferior facet angle (AIFA), ASFS, and ASFEA in Ctrl-P significantly differed from OO-AD.C0-C1, C1-C2, C0-C2, C2-C7, C2-C7 SVA, and C2S in Ctrl-P significant differed from the OO-AD group. C2-C7 SVA and C2S in Ctrl-N significantly were smaller than the OO-PD group. C1-C2 correlated with C0-C1 and C2-C7 negatively in the OO group. Slight kyphosis of C1-C2 in OO-AD was compared with lordosis of C1-C2 in Ctrl-P, inducing increased extension of C0-C1 and C2-C7. Mildly increased lordosis of C1-C2 in OO-PD was compared with C1-C2 in Ctrl-N, triggering augmented flexion of C0-C1 and C2-C7.

**Conclusion:**

ASFS was vital in determining directions of OO with AAD and explaining compensations. ASFS and ASFEA could provide pre- and intraoperative guidelines.

**Key Points:**

*• ASFS may determine the directions and compensatory mechanisms of AAD secondary to OO.*

*• ASFS could be achieved by the sum of ASFEA and C2S.*

## Introduction

The aetiologies of atlantoaxial displacement (AAD, including instability, subluxation and dislocation) include trauma, inflammation, and genetics, among which os odontoideum (OO) is an important factor [1]. Although OO triggers AAD, which is characterised by higher morbidities of anterior displacement (AD) than posterior displacement (PD), by regulating lateral atlantoaxial joints (LAJs) [2–4], the elements determining displacement directions are still unclear.

The atlantooccipital complex can serve as the moving object during AAD based on the recognition of the axial superior facet (ASF) as the sliding surface. While few reports have focused on ASF, it is vital to explore ASF characteristics based on significance by comparing AD with PD.

Nontraumatic L5 spondylolisthesis features forwards translation and rotational displacement of L5 vertebrae on the S1 upper endplate, while posterior sliding of L5 is seldom mentioned (except for the loss of L5/S1 lordosis and retrolisthesis in slump-sitting posture [5]). The reason for this is the orientation of the upper sacral endplate inclined from anteroinferior to posterosuperior in the upright position [6]. This made us consider whether the displacement is related to the anatomy and position of the ASF in the upright position. Therefore, the axial superior facet endplate angle (ASFEA), an anatomical parameter derived from supine cervical CT, and the axial superior facet slope (ASFS) were proposed to explore the relationships of AAD directions to recognize the ASF in standing spinal X-ray examinations and limit the use of standing cervical CT examinations.

Hitherto, their little attention has been given to the regulation of cervical-global sagittal balance [7], especially compensations of the atlantooccipital joint and subaxial cervical spine after AAD. However, whether AAD can trigger compensations as does spondylolisthesis remains unknown. Herein, we found that the ASFS might determine the displacement orientation of AAD secondary to OO and trigger compensations between the atlantooccipital joint and subaxial cervical spine, thereby providing guidelines for treating AAD.

## Methods

### Data collection

Research was performed on patients admitted from 2011 to 2021 undergoing surgery for upper cervical myelopathy caused by OO with AAD. Patients who underwent surgery for spinal compression caused by OO with AAD and who had clear radiographical data were included. Patients with AAD caused by trauma, rheumatoid arthritis (RA), inflammation, infection, tumour, history of surgery on the cervical spine and OO combined with atlas occipitalisation were excluded. Twenty-four patients diagnosed with OO with AAD were included in the OO group. Seventy-two patients with cervical degeneration without an occipitocervical disorder were included in the control group (Ctrl) at a 1:3 ratio with matching by age and sex to the OO group. Patients in the Ctrl group had cervical disc degenerative diseases with supine cervical CT data, standing cervical lateral radiographs and no occipitocervical abnormalities.

### Measurements of cervical sagittal parameters

**Fig. 1:**
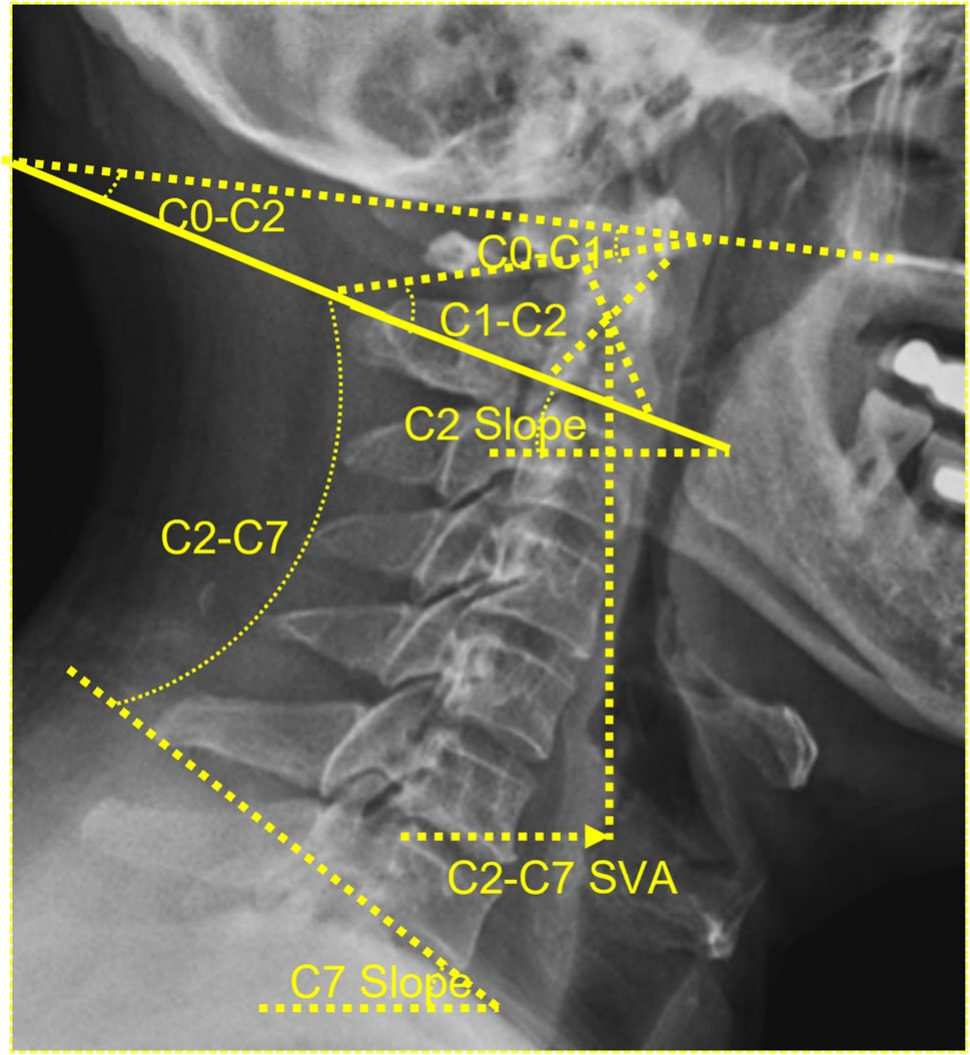
Measurement of radiographical parameters on X-ray examination

Cervical sagittal parameters are shown in Table 1 and Fig. 1. C7S can be used to replace T1S to avoid an unclear upper T1 endplate [8, 9].

**Table 1:** Cervical parameters

Title	Definition	Radiographic data
C0-C1 cobb angle	C0-C1 cobb (C0-C1) angle was measured as the angle between McGregor’s line with the tangent line of the atlas inferior edge	X-ray
C1-C2 cobb angle	C1-C2 cobb angle (C1-C2) was the intersection angle between the line of the lower point of the anterior and posterior tubercle of the atlas and the line parallel to the lower endplate of C2	X-ray
C0-C2 cobb angle	C0-C2 cobb angle (C0-C2) was between McGregor's line and axial lower endplate	X-ray
C2-C7 cobb angle	C2-C7 cobb angle (C2-C7) was between the axial lower endplate and C7 lower endplate	X-ray
C0-C7 cobb angle C2-C7 SVA	C0-C7 cobb (C0-C7) angle was measured as the angle between McGregor's line and C7 lower endplate C2-C7 SVA was the distance between the gravity line of the center of C2 and posterosuperior margin of C7	X-ray X-ray
C2 slope	C2 Slope (C2S) was the angle between the axial lower endplate with horizontal line	X-ray
C7 Slope	C7 Slope (C7S) was the angle between lower endplate of C7 and horizontal line	X-ray
Sagittal inferior C1 facet angle	Sagittal inferior C1 facet angle (AIFA) referred to previous study and it was between the line connecting the anteroinferior edge of the lateral mass and the upper edge of the posterior arch of the atlas and the line connecting the two points at the edge of C1 articular surface of lateral mass joint [20]	CT
ASFEA	Seen in “Materials and methods”	CT
ASFS	ASFS was between the line connecting the anterior and posterior points of ASF and horizontal line, which was equal to the sum of C2S + ASFEA	X-ray and CT

Definitions and different radiographic data sources required for the determination of cervical parameters are listed to clarify the measurement procedures

**Fig. 2:**
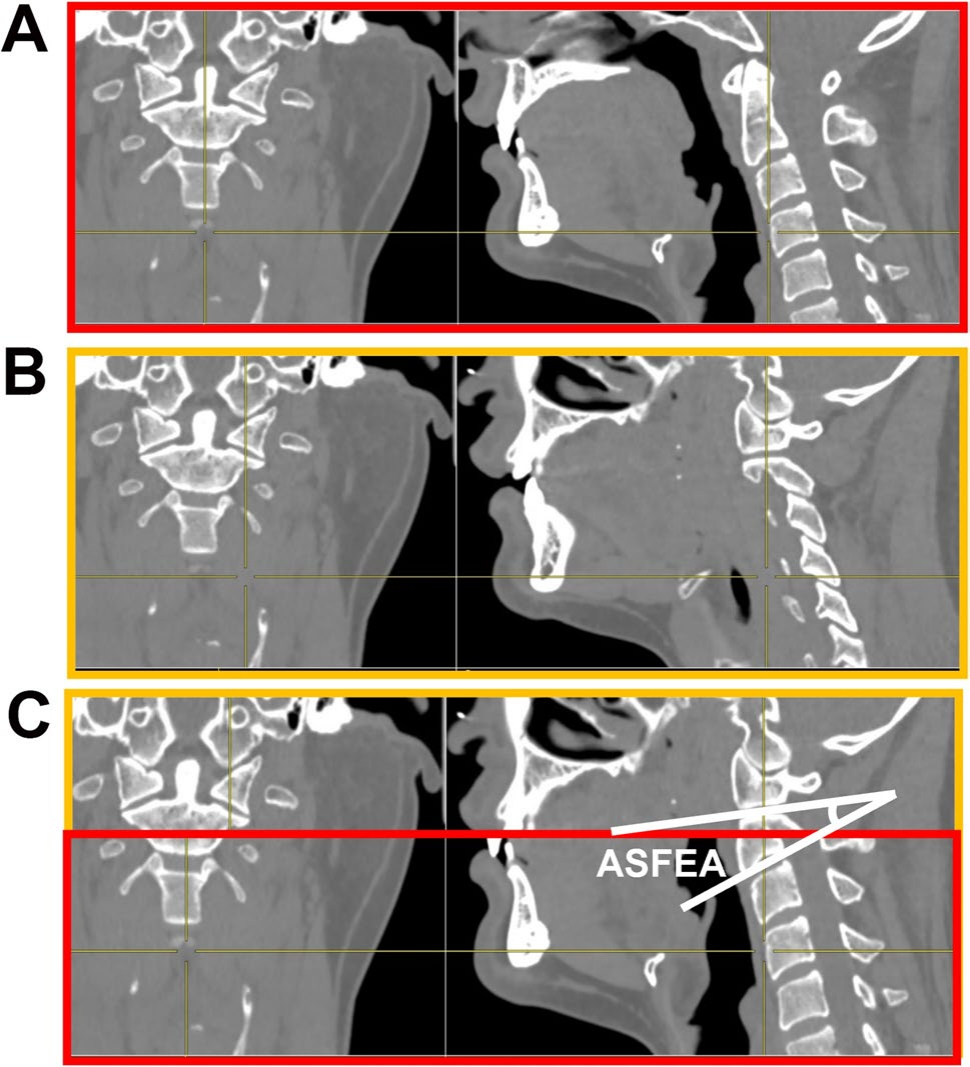
Method for ASFEA measurement. **A** Mid-sagittal CT reconstruction of the axis (red frame) and ASF (orange frame) (**B**) were obtained. **C** Two images were aligned, overlapped and cropped to demonstrate the axial inferior endplate and ASF simultaneously. The ASFEA was the angle between the line connecting the anterior and posterior points of the ASF and the extension line of the axial lower endplate

**Fig. 3:**
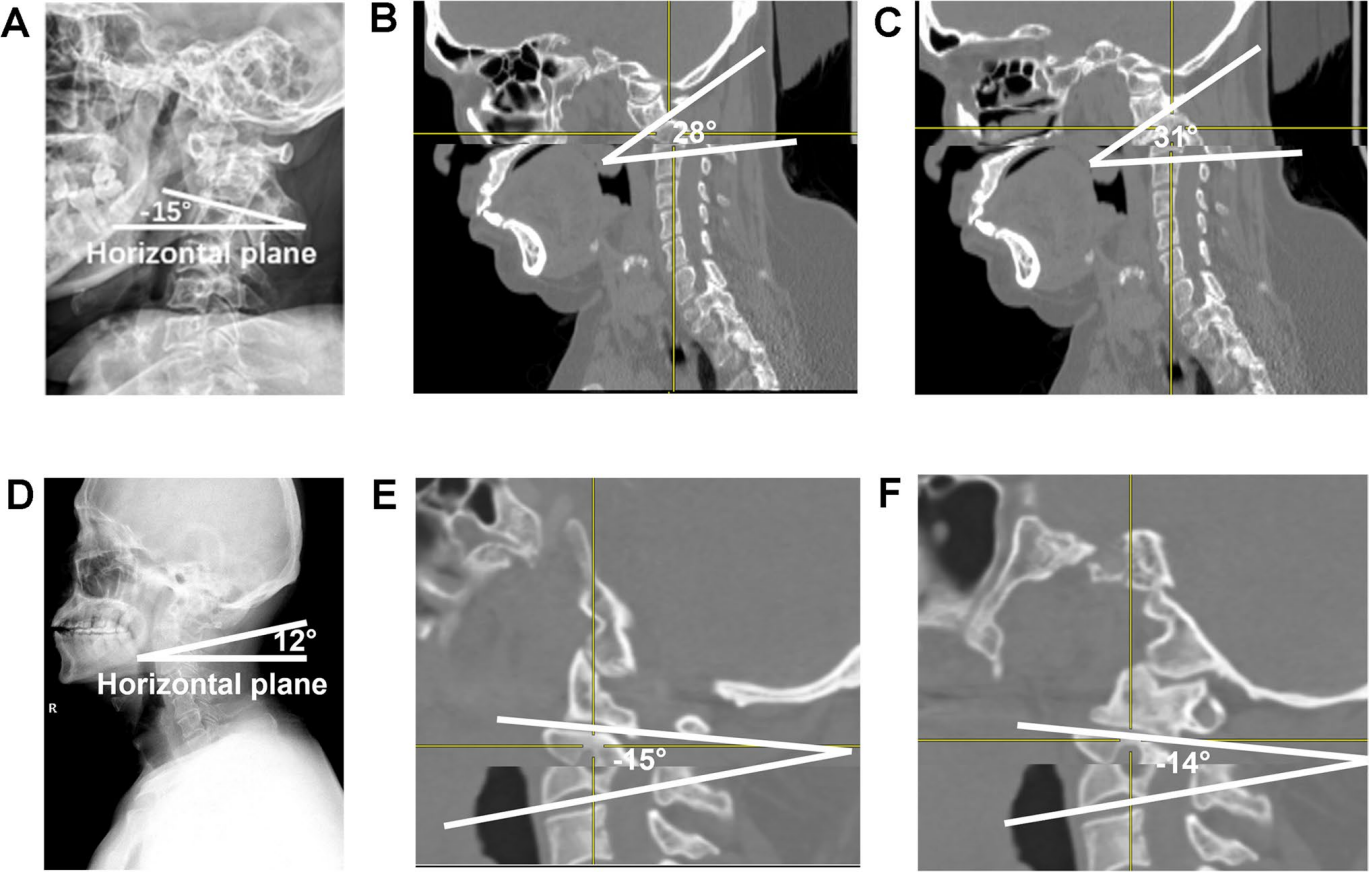
Measurement of ASFEA in AAD patients. Herein, C2S + ASFEA = ASFS. **A** Cervical X-ray of an AD patient in a neutral position and measurement of C2S. **B** Measurement of left ASFEA in an AD patient. **C** Measurement of right ASFEA in an AD patient; (left ASFEA (28°) + right ASFEA (31°))/2 + C2S (− 15°)) = ASFS (14.5°). **D** of Cervical X-ray of a PD patient in a neutral position and measurement of C2S. **E** Measurement of left ASFEA in a PD patient. **F** Measurement of right ASFEA in a PD patient; (left ASFEA (− 15°) + right ASFEA (− 14°))/2 + C2S (12°)) = ASFS (− 2.5°)

ASFEA measurements are shown in Fig. 2, and the specific steps are as follows: (1) Mid-sagittal CT reconstructions of the axis and ASF were captured. (2) Two images were aligned, overlapped and cropped according to positioning lines such that the final image clearly demonstrated the axial inferior endplate and ASF simultaneously. (3) ASFEA was measured as the angle between the line connecting the anterior and posterior points of the ASF and the extension line of the axial lower endplate. In addition, C2S was measured, and ASFS was obtained as the sum of ASFEA and C2S (Fig. 3).

When the angle vertex lay in front of or behind the cervical spine, the values were positive or negative, respectively. Positive values of C0-C1, C1-C2, C0-C2, C2-C7, C0-C7, and ASFEA indicated kyphosis, and negative values indicated lordosis. Positive values of C2S, C7S, and ASFS implied that the lower endplate of C2, C7, and ASF were inclined from anteroinferior to posterosuperior compared with the horizontal plane (AIPS), while negative values represented inclination from anterosuperior to posteroinferior (ASPI).

### Consistency analysis

Two surgeons measured and calculated ASFS independently every 2 weeks. Mean measurements were used for further accuracy. Intraobserver and interobserver reliability were analysed.

### Statistical analysis

Data were analysed using SPSS 28.0 (SPSS, Inc.). Parameters in the two groups were compared by *t* tests. Reliability was calculated by the intraclass correlation coefficient (ICC) [10]. Correlation analysis was performed using the Pearson method. A *p* value < 0.05 was considered to indicate statistical significance.

## Results

### Demographic information

The OO group included 14 men and 10 women, aged 51.13 ± 13.50 years, as well as 20 OO-AD cases (83.3%) and 4 OO-PD cases (16.7%). The Ctrl group included 43 men and 29 women, aged 45.79 ± 20.29 years; there were four cervical sagittal curve types in the Ctrl group, including 43 lordosis cases (59.7%), 16 neutral cases (22.2%), 8 kyphosis cases (11.1%), and 5 S-shaped cases (7.0%) according to a previous report [11].

### Reliability analysis of ASFS

Table 2 shows that the intraobserver and interobserver reliability were both excellent for ASFS.

**Table 2:** Reliability analysis of ASFS

Measurement	Statistical indexes (M ± SD)	Intra-observer reliability	Inter-observer reliability
A1	3.79 ± 9.09°	0.863 (*p* < 0.001)	0.866 (*p* < 0.001)
A2	3.99 ± 8.23°		
B1	4.70 ± 7.87°	0.929 (*p* < 0.001)	0.939 (*p* < 0.001)
B2	4.15 ± 9.02°		

Measurements were obtained by two examiners twice at an interval of 2 weeks and are listed as A1, A2 and B1, B2, respectively. Intraobserver and interobserver reliability were calculated to assess the reliability of the ASFS

### ASFEA, sagittal inferior C1 facet, and ASFS

ASFEA, ASFS and AIFA are shown in Table 3.

**Table 3:** Parameters of Atlantoaxial lateral mass

	Ctrl-P	OO-AD	*p* value	Ctrl-N	OO-PD	*p* value
ASFEA	− 10.98 ± 5.35°	15.78 ± 12.16°	< 0.001	− 17.42 ± 5.07°	− 21.67 ± 4.04°	0.173
ASFS	8.75 ± 4.65°	13.24 ± 6.99°	< 0.001	− 7.13 ± 2.41°	− 4.27 ± 2.35°	0.06
AIFA	12.01 ± 3.00°	7.93 ± 3.33°	< 0.001	14.88 ± 3.02°	17.48 ± 1.40°	0.156

Parameters of atlantoaxial lateral mass, including ASFEA, ASFS and AIFA, were measured in different groups to determine morphological and functional changes. ASFEA and AIFA were measured to determine morphological changes in the C1 and C2 facets in the different groups. ASFS was measured as the axial functional parameter in various groups

Positive ASFS values indicated AIPS of the ASF, and the atlantooccipital complex tended to slide forwards; conversely, it tended to slide backwards. OO-AD was compared with Ctrl-P (positive, ASFS > 0), and OO-PD was compared with Ctrl-N (negative, ASFS < 0) to analyse the inferior C1 facet and ASF morphology.

The OO group included 19 positive ASFEA cases (79.2%) and 5 negative cases (20.8%). All cases in the Ctrl group had positive ASFEA values (100%); the OO group included 20 positive ASFS cases (83.3%) and 4 negative ASFS cases (16.7%). In the neutral position, the Ctrl group had 57 positive ASFS cases (79.2%) and 15 negative ASFS cases (20.8%). In the overflexion position, the Ctrl group had 68 positive ASFS cases (94.4%) and 4 negative ASFS cases (5.6%).

### Compensations of the atlantooccipital joint and lower cervical spine for OO-AD and OO-PD

Comparisons of cervical sagittal parameters are shown in Table 4, and the correlation analysis results are shown in Table 5.

**Table 4:** Comparisons of cervical sagittal parameters

	Ctrl-P	OO-AD	*p* value	Ctrl-N	OO-PD	*p* value
C0-C1 C1-C2 C0-C2	5.98 ± 8.94° − 27.20 ± 8.62° − 20.69 ± 9.19°	− 3.80 ± 5.06° 6.14 ± 8.97° 2.35 ± 7.37°	< 0.001 < 0.001 < 0.001	10.69 ± 3.90° − 28.21 ± 5.06° − 17.53 ± 5.55°	11.97 ± 3.55° − 33.07 ± 9.50° − 21.10 ± 7.33°	0.592 0.156 0.310
C2-C7 C0-C7 C2S C7S	0.73 ± 13.20° − 20.75 ± 13.05° 19.65 ± 6.20° 23.96 ± 8.84°	− 28.44 ± 12.72° − 26.09 ± 12.35° − 3.43 ± 10.01° 23.72 ± 9.14°	< 0.001 0.145 < 0.001 0.924	− 10.40 ± 6.51° − 27.93 ± 10.48° 10.23 ± 3.34° 28.86 ± 6.07°	− 8.10 ± 3.57° − 26.20 ± 4.24° 17.40 ± 2.52° 33.23 ± 2.53°	0.556 0.838 < 0.001 0.055
C2-C7 SVA	23.89 ± 11.22 mm	16.99 ± 7.90 mm	0.019	18.04 ± 4.78 mm	38.27 ± 6.15 mm	< 0.001

Cervical sagittal parameters in the Ctrl and OO groups measured on X-ray examination according to the definitions are listed in the table. Parameters were compared between the Ctrl-P and OO-AD groups and the Ctrl-N and OO-PD groups to further assess changes in cervical sagittal curves

**Table 5:** Correlation Analysis

	C1-C2	C0-C1	C2-C7	C2S	C2-C7 SVA	C7S
OO group						
C1-C2	−	− 0.781**	− 0.636**	− 0.831**	− 0.550*	− 0.334
C0-C1	− 0.781**	−	0.512*	0.580**	0.416*	0.110
C2-C7	− 0.636**	0.512*	−	0.788**	0.420*	− 0.372
C2S	− 0.831**	0.580**	0.788**	−	0.614**	0.159
C2-C7 SVA	− 0.550*	0.416*	0.420*	0.614**	−	0.394
vC7S	− 0.334	0.110	− 0.372	0.159	0.394	−
OO-AD group						
C1-C2 C0-C1 C2-C7 C2S	− − 0.570** − 0.451* − 0.642**	− 0.570** − 0.305 0.264	− 0.451* 0.305 − 0.702**	− 0.642** 0.264 0.702** −	0.087 0.017 0.097 0.262	− 0.061 − 0.163 − 0.702** − 0.141
C2-C7 SVA C7S	0.087 − 0.061	0.017 − 0.163	0.097 − 0.702**	0.262 − 0.141	− 0.202	0.202 −
OO-PD group						
C1-C2 C0-C1 C2-C7	− − 0.634 − 0.749	− 0.634 − − 0.038	− 0.749 − 0.038 −	− 0.894 0.219 0.967	0.038 − 0.797 0.634	− 0.314 − 0.535 0.864
C2S C2-C7 SVA C7S	− 0.894 0.038 − 0.314	0.219 − 0.797 − 0.535	0.967 0.634 0.864	− 0.415 0.707	0.415 − 0.937	0.707 0.937 −

Correlations among C1-C2, C0-C1, C2-C7, C2S, C2-C7 SVA, and C7S in different OO groups were calculated to determine the compensatory changes in different cervical segments after displacement. C0-C1, C1-C2, C2-C7, C2S, C7S = (°); C2-C7 SVA = (mm); * represents *p* value < 0.05, **represents *p* value < 0.01

Comparison between the OO-AD and Ctrl-P groups demonstrated that the former had significantly larger C1-C2 and C0-C2 and smaller C0-C1, C2-C7, C2-C7 SVA and C2S values, with no significant differences in C7S or C0-C7 values. Comparison between the OO-PD and Ctrl-N groups demonstrated that the former had significantly larger C2S and C2-C7 SVA values, with no significance in C0-C1, C1-C2, C0-C2, C2-C7, C0-C7, and C7S values.

Correlation analysis showed that C1-C2 correlated positively with C0-C2; C0-C1, C2-C7, C2-C7 SVA, C2S, and C7S values correlated positively with each other except for a weak negative correlation between C2-C7 and C7S; and C1-C2 and C0-C2 correlated negatively with C0-C1, C2-C7, C2-C7 SVA, C2S, and C7S values. The weakest correlations were in the OO-PD group, possibly due to sample size limitations, nonobvious compensations and slight PD. Strong correlations were observed in the OO-AD group, possibly due to sufficient sample size, thus inducing apparent compensations. The strongest correlations were observed in the OO group.

## Discussion

AAD has been described in diseases such as traumatic odontoid fracture, OO, and RA [12], featuring higher morbidity in AD than in PD. Morphologic remodelling of axial LAJs during AAD has attracted much attention. LAJs with non-union odontoid fractures gradually remodel into a fish-lip or dome-shape during AAD, resulting in irreducibility [13, 14]. Salunke found that the AIFA was linked with disease progression and irreducibility [15]. Ma also classified the morphology of lateral C1-C2 joint facets in congenital AAD patients based on the AIFA [16]. These results reflect the atlantoaxial facet morphology but fail to consider OO patients and positional parameters of atlantoaxial facets.

In AAD, the ASF is the sliding surface, while the atlantooccipital complex is the sliding object. Hence, the ASF better reflects the mechanisms of AAD. A previous study assessed the atlantoaxial superior facet morphology in AAD and showed the significance of sagittal joint inclination, similar to ASFEA, in determining AAD severity [17]. However, it was more anatomical than positional. Yuan proposed a novel cervical parameter, sagittal atlantoaxial joint inclination (SAAJI), illustrating the atlantoaxial articular surface on the parasagittal view for irreducible AAD, which resembled ASFS [18]. However, horizontal lines were difficult to determine on supine CT, which restricted its clinical application. Additionally, the sagittal slippage angle of the atlantooccipital inferior joint facet uses the eye-ear plane as the horizontal plane, which fails to reflect real conditions while standing [19]. In addition, the specificity of patients and the ASF were not mentioned.

These studies focused on morphologic changes in LAJs after long-term remodelling in AAD while neglecting initial factors propelling AD rather than PD. A previous study revealed that PD was usually found with odontoid destruction in RA [20]. Mechanisms of irreducible PD with OO showed that once the C1 facet posteriorly crosses the medial hump of the C2 facet, it tends to slide further and locks in this position, making the PD irreducible. Theoretically, the odds of AD should equal those of PD, which is in contrast to the clinical observation that AD cases outnumber PD cases.

The displacement in patients with OO-related fracture or RA is accidental to some extent based on the trauma to or pathological destruction of the atlantoaxial joints. Thus, only in cases of OO and type II transverse odontoid fracture without obvious displacement would the atlantoaxial vertebrae experience a long process ranging from instability and subluxation to irreducibility, during which the displacement of the atlas compared with the axis and the remodelling of LAJs advance together and have mutual effects. When traced back to the initial process of displacement, however, the facet surface of LAJs showed no significant changes, but AD was obviously more prevalent than PD, prompting us to consider the relationship between ASFS and the direction of AAD.

To determine the cause-effect relationship between ASFS and OO diagnosed with AAD, it was advisable to conduct a cohort study for the measurement of ASFS before displacement and analyse their correlation. However, these patients are normally diagnosed with neurologic symptoms after AAD, or even if they are occasionally diagnosed in the early stage, surgery is immediately recommended to avoid neurologic sequelae [1], making it difficult to collect cases from prospective cohort observations. The alternative was to study the ASFS in people without AAD and compare it with the ASFS in patients with OO with AAD to indirectly understand the relationship between the ASFS and the direction of AAD in OO. Patients with cervical disc degenerative disease were included in the Ctrl group, which could better reflect the true conditions under physiological circumstances. Therefore, we studied patients with and without AAD to indirectly demonstrate the crosstalk between ASFS and displacement directions.

Herein, ASFEA and ASFS were consequences of AAD after remodelling, which cannot represent the initial conditions of the atlantoaxial facet surfaces. The displacement of the atlantooccipital complex on the ASF and the remodelling of LAJs have mutual influences, which cannot be interpreted as only causes or results [3, 21–23]. Moreover, their relationship should be recognised as an interaction, as one condition could significantly affect the other while also accelerating the displacement process. Since it was impossible to track ASFEA and ASFS before AAD, atlantoaxial parameters from non-AAD patients were used as a substitute approach to reflect them before AAD. Notably, the skeletal system remains steady in adults, leading to presumptions that ASFEA and ASFS stay constant in adults, which corresponded to pelvic inclination (PI) and neutral sacral slope (SS) in a previous report [24]. OO patients were an average of 51.13 ± 13.50 years old (we were not a specialised children’s hospital), suggesting that AAD might start following adulthood. Therefore, it was better to measure ASFS and ASFEA in the non-AAD group to simulate conditions before AAD. In this study, the displacement in OO patients (AD (20 cases, 83.3%), PD (4 cases, 16.7%)) was consistent with that in previous reports [1, 4]. In the control group (neutral), a positive ASFS was found in 57 (79.2%) patients, and a negative ASFS was found in 15 (20.8%), whereas a positive ASFS was found in 68 (94.4%) patients on hyperflexion radiography. These findings indicate that the distribution of displacement in OO was approximate to that of the ASFS in the Ctrl group, underlying the potential clinical significance of ASFS for determining displacement direction in OO patients.

Specifically, when the ASFS was 0° in OO patients before AAD, the ASF remained relatively horizontal, maintaining the stability of the LAJs and surrounding structures. Less mechanical loading would be applied to LAJs in the absence of remodelling. However, a positive ASFS would enable the tendency to slide forwards given that the facet surfaces were AIPS based on gravity. In turn, LAJs with AD would result in further remodelling of facet surfaces to increase the ASFS and contracture of periarticular stabilisers to induce abnormalities of LAJs, leading to anterior instability, subluxation, and luxation of the LAJs [19]. Conversely, a negative ASFS tended to slide backwards under gravity for ASPI.

Most AAD patients were AD with a positive ASFS and PD with a negative ASFS, leading to a higher prevalence of AD than PD, which was aggravated by head-lowering movements during daily work [25]. Kauppi reported that during neck hyperextension, the contact between the posterior arch of the atlas and the spinous process restrained PD, while such restriction did not completely work during flexion, partially explaining the lower incidence of PD [26]. We further illustrated that AAD initiated even in a neutral position, possibly due to ASFS and ASFEA imbalances.

Furthermore, sacral osteotomy has been reported in the correction of pelvic parameters (PI) for maintaining spinal-pelvic sagittal balance [27], hinting at possibilities of adjusting the ASFS via ASF osteotomy or C2-C3 fusion, thereby preventing AAD and preserving atlantoaxial functions without spinal syndromes. Osteotomy on the inferior C1 facet and ASF could convert AAD irreducibility and thereby independently realise an intraoperative reduction of LAJs by a posterior approach [15]. This further shows the significance of the ASFEA and ASFS intraoperatively; otherwise, the opposite osteotomy may be performed, which would aggravate the irreducibility of AAD or deteriorate the deformity of LAJs. Generally, the ASFS and ASFEA could help surgeons adjust treatments appropriately in early stages to delay or reverse AAD progression, especially to facilitate the osteotomy direction based on the ASFEA [18, 28]. More importantly, the ASFS and ASFEA predicted the direction of AAD, which was useful for instructing patients to adjust their neck posture and effectively suspend displacement.

Lordosis of the ASFEA became kyphosis with an average of 25°, and the AIFA decreased by approximately 5° due to remodelling after OO-AD. The ASFS in OO-AD increased, which further explained the crosstalk between the ASF and displacement direction. Atlantoaxial parameters in the comparison between the Ctrl-P and OO-PD groups were not significantly different, possibly due to the sample size and blockage of the odontoid process.

Notably, sagittal imbalance of the cervical spine has been shown to induce compensations in adjacent segments. Cervical parameters have been used to analyse sagittal compensations of the cervical spine, such as in C0-C2, C1-C2, C2S, and T1S [29, 30], and crosstalk between C2S and cranial slope (CS) has been reported in the superior cervical spine [7]. Plain standing spinal films enabled the measurement of C2S, propelling us to further determine ASFS and ASFEA. We showed that ASFS = C2S + ASFEA, where ASFEA was an anatomical parameter that remained stable regardless of position, indicating a positive correlation between ASFS and the functional parameter C2S. The results showed that both ASFS and C2S correlated with C1-C2, C0-C2, and C2-C7, indicating that AIPS facets of a positive ASFS induced larger C1-C2 and smaller C0-C1 and C2-C7 values in AD patients, while ASPI facets of a negative ASFS induced smaller C1-C2 and larger C0-C1 and C2-C7 values in PD patients, thereby maintaining balance during standing. A larger ASFS in AD patients led to lordosis of C0-C2 and C2-C7, showing that craniocervical compensations were involved in maintaining a horizontal view. However, patients with an excessively large ASFS developed severe cervical lordosis in the late stages of AD, which triggered unsustainable compensations, leading to failure to maintain a horizontal gaze while standing.

To maintain an upright standing posture and horizontal gaze, the spine as a whole system is balanced by adjusting the spinal segments. Consequently, the musculoskeletal system coordinates spinal alignment to adjust physiologic curves and maintain a horizontal view [31]. Compensations in adjacent vertebrae usually arise when spinal misalignment appears in localised segments [32]. Herein, sagittal deformity of C1-C2 led to compensations of C0-C1, C2-C7, C2-C7 SVA, C2S, and C7S, indicating that sagittal parameters exerted apparent effects on adjacent segments but minor impacts on distal segments. A negative C1-C2 in Ctrl-P represented lordosis, while a positive C1-C2 in OO-AD indicated kyphosis, with an approximate difference of 33°. Changes in the C0-C1 and C2-C7 curves indicated compensations of approximately 10° and 30°, respectively, driven by C1-C2 kyphosis, which further resulted in C2-C7 SVA and C2S decreases. No significance was found in C7S, showing that the thoracolumbar spine was not involved in compensations for C1-C2 deformity. Significance was found in C2-C7 SVA and C2S when OO-PD was compared to Ctrl-N, indicating that OO-PD patients developed slight morphologic alterations of atlantoaxial joints, inducing compensations in atlantoaxial adjacencies. However, this finding is carries less meaning considering the sample size of OO-PD patients.

Understanding C1-C2 deformity and compensations in AAD can help surgeons adjust fusion angles intraoperatively. Although the reported fusion angle was 20–22° for AAD with OO, another study argued that it was unnecessary to recover the C0-C2 fusion angle to the normal range intraoperatively [33, 34]. Herein, a horizontal gaze was maintained by C2-C7 adjustments due to the absence of C0-C1 compensations in C0-C2 fusion. The fixation angle of C0-C2 should be emphasised to avoid lower cervical spine degeneration. In contrast, the C1-C2 fixation angle allowed a larger range to maintain a horizontal view after C1-C2 fusion by adjusting C0-C1 and C2-C7 to compensate for C1-C2 deformity. Limitations of this study include the sample size and inherent limitations of cohort studies. Therefore, relationships between the ASFS and the direction of AAD in OO patients remain speculative.

## Conclusions

The ASFS and ASFEA were introduced for determining the direction of AAD. A positive ASFS indicated AIPS, suggesting the tendency of AD due to forwards sliding, while a negative ASFS indicated ASPI in PD. Compensations after displacement included OO-AD inducing increased flexion of C0-C1 and C2-C7 and decreased flexion of C1-C2; OO-PD triggered opposite compensations. Therefore, recognition of the ASFS and ASFEA provided referential guidelines pre- and intraoperatively.

## Abbreviations

AAD: Atlantoaxial displacement
AD: Anterior displacement
AIFA: Atlas inferior facet angle
AIPS: Incline from anteroinferior to posterosuperior compared with the horizontal plane
ASF: Axial superior facet
ASFEA: Axial superior face endplate angle
ASFS: Axial superior facet slope
ASPI: Incline from anterosuperior to posteroinferior compared with the horizontal plane
C2S: C2 slope
C7S: C7 slope
LAJ: Lsateral atlantoaxial joint
OO: Os odontoideum
PD: Posterior displacement

## Declarations

### Guarantor

The scientific guarantor of this publication is Prof. Changqing Zhao.

### Statistics and biometry

No complex statistical methods were necessary for this paper.

### Methodology

• Retrospective
